# Do work- and home-related demands and resources differ between women and men during return-to-work? A focus group study among employees with common mental disorders

**DOI:** 10.1186/s12889-020-10045-4

**Published:** 2020-12-17

**Authors:** Lotta Nybergh, Gunnar Bergström, Therese Hellman

**Affiliations:** 1 Unit of Intervention and Implementation Research for Worker Health, Institute of Environmental Medicine, Karolinska Institutet, Nobels Väg 13, Box 210, SE-171 77 Stockholm, Sweden; 2 Department of Occupational Health Sciences and Psychology, University of Gävle, 801 76 Gävle, Sweden; 3 Occupational and Environmental Medicine, Department of Medical Sciences, Uppsala University, Uppsala University Hospital, 751 85 Uppsala, Sweden

**Keywords:** Return-to-work, Common mental disorders, Focus group, Qualitative study, Gender, Home-related demands, Work-related demands, Sweden

## Abstract

**Background:**

Common mental disorders present the main reason for registered sick leave in Sweden today, and women are at a higher risk of such sick leave than men. The aim of our study was to explore how the experiences of work- and home-related demands as well as resources influence return-to-work among employees sick-listed for common mental disorders in Sweden. Specifically, we aimed to explore similarities and differences in patterns of experiences among women and men.

**Methods:**

A qualitative design with semi-structured focus group interviews was applied. One pilot interview and six additional focus groups, with a total of 28 participants, were conducted. The focus group discussions were audiotaped and transcribed verbatim. Data was analyzed with conventional content analysis.

**Results:**

The analysis resulted in four main categories and eight sub-categories. While the study aim was to explore aspects of work and home, additional considerations related to internal demands and involved actors were also found. The main and sub-categories were “Home-related demands and resources” (sub-categories: “Not on sick leave for home-related demands”, “Feeling responsible for relationships and the well-being of others”, “An affected economy” and “Finding energizing activities and creating routines”), “Work-related demands and resources” (sub-categories: “Encountering tough emotions and an over-bearing feeling of responsibility at work”, “Continued work-related demands create un-certainty about the future”, “Loss of boundaries” and “(Desired) support from managers and colleagues”), “Internal demands and resources” and “Demands and resources linked to involved actors”. The experiences described among women and men were similar in some categories while patterns of experiences differed in others.

**Conclusions:**

Home-related demands and resources influence return-to-work among women and men sick-listed for common mental disorders in Sweden, also when work-related demands are experienced as the main reason for the sick leave period. Furthermore, several of these aspects were described differently among women and men, which highlights the need to consider possible gender differences in relation to return-to-work, while maintaining attention to individual variations.

**Supplementary Information:**

The online version contains supplementary material available at 10.1186/s12889-020-10045-4.

## Background

Common mental disorders (CMDs) such as depression, anxiety and stress-related disorders present the main diagnosis for registered sick leave in Sweden today [1]. CMDs are also highly prevalent in other OECD countries [2] and even globally, despite regional variability [3]. Moreover, women are at a higher risk of being on CMD-related sick leave than men. Out of those sick-listed in Sweden in 2017, 53% of the women and 41% of the men were sick-listed for a CMD-related diagnosis [1], a discrepancy that has widened since the 1980’s [4]. Being on sick leave for mental ill health affects the employee’s identity, self-respect and general well-being [5]. It also poses a risk for social and professional isolation in the long-term [6], as well as economic loss at the individual, employer and societal levels [2]. These effects make the employee’s return-to-work (RTW) an important issue to study.

Several reasons, which could have implications for the RTW process, have been proposed to explain the difference in prevalence of CMD-related sick leave between women and men. One often cited explanation is that working conditions in female dominated occupations are characterized by factors known to increase the risk for sickness absence due to mental ill health, such as high demands, low control, and imbalance between effort and reward. Moreover, men are more frequently in leading positions in both male and female dominated professions with greater opportunities for wage increases and greater control over their own work situation [7]. Another aspect relevant to RTW is gender norms and hierarchies, which are known to influence work and health. For instance, some studies have found that women have difficulties in drawing boundaries at work [8, 9]; the women in one of these studies felt that drawing boundaries was socially unacceptable, stemming from a perception of having lower social status [9]. A review of gender research on work environment and work organization studies also found that women’s social subordination, in combination with difficulties in setting limits and a poor reliance in one’s own capabilities, affects women’s work health negatively [7]. Further, norms related to masculinity may reduce men’s inclination to seek help for health-related concerns [10], especially those related to mental ill health [11].

Additional factors that have been proposed to explain the sex discrepancy in sick leave rates due to mental ill health include stresses imposed by combining home-related demands with work-related ones. These have especially been studied with regards to taking care of children, elders and relatives with special needs. However, the evidence is inconclusive [12]. One study noted that although employees, supervisors and occupational physicians considered home/work balance as one of the most important aspects of RTW, this aspect is seldom investigated among current studies on RTW [13]. A recent paper, which presented an integrated framework for sustainable RTW among employees with CMDs, similarly highlighted the need for considering both the work and home domains to fostering or hindering sustainable RTW [14]. Some have also looked at role overload theory which assumes that multiple roles may have negative effects on sick leave and health, and its counterpart role enhancement theory, which presumes positive health effects of multiple roles [15, 16].

Three literature reviews of quantitative studies on factors influencing RTW among employees with mental ill health found inconclusive evidence on the effect of gender on personal and work-related factors [17–19]. One of the reviews called for further research on how gender may affect RTW for employees with mental ill health, as such aspects have been less studied [19]. The reviews did not find or mention factors related to the home, which may reflect that they are generally less studied in relation to RTW than work-related factors [20]. Furthermore, most studies that have considered both work- and home-related aspects have focused on predicting CMD-related sick leave, and fewer have considered their influence on RTW [21]. Finally, most studies that include home-related aspects focus on women and only a few compare women with men.

Quantitative studies are well-suited to investigate predictors for RTW and to evaluate the impact of RTW interventions. However, qualitative studies are required to explore the complexities of the RTW process and to illuminate how the affected individuals themselves experience demands and resources related to RTW [22]. A qualitative study design is also valuable for exploring how gender norms may influence experiences related to RTW. A meta-review of qualitative studies of employees sick-listed for CMDs found that obstacles and facilitators related to their own personality, social support at the workplace, and the social and rehabilitation systems influenced RTW [22]. However, the review found only a few qualitative studies on this subject and called for additional interview-based studies. The review did not mention home-related demands or resources, nor did it evaluate whether the results may have differed for male and female participants. One study that interviewed women and men in Sweden on sick leave for musculoskeletal disorders found similarities as well as differences between the factors in the private arena that affected RTW [23]. The authors argued that domestic strain, i.e. inequities in the division of work tasks and responsibilities, and isolation and lack of socio-emotional support at home, hindered RTW among the interviewed women. When it comes to CMD-related sick leave, little is known about employees’ experiences of combined work- and home-related demands as well as resources in relation to RTW, and especially whether women’s and men’s experiences might differ.

The aim of our study was to explore how the experiences of work- and home-related demands as well as resources influence return-to-work among employees sick-listed for common mental disorders in Sweden. Specifically, we aimed to explore similarities and differences in patterns of experiences among women and men.

## Methods

The consolidated criteria for reporting qualitative research are followed in this paper [24].

### Design

A qualitative design with semi-structured focus group interviews was applied. The interviews were designed to answer two distinct but intersecting aims, of which the first (on the experiences of demands and resources during RTW) is presented in the current manuscript and the other (on the experiences of interventions and rehabilitation activities) in a forthcoming paper. As the subject is relatively less explored, the usage of focus groups was chosen to generate a variety of perspectives [25]. It was assumed that the interaction among the participants would help to explore and clarify the participants’ experiences and views in relation to the study aims.

### Data collection and participants

The current study is part of a larger project that aims to evaluate an intervention by the occupational health services (OHS) directed at employees with CMDs or stress-related symptoms at work [26]. A participant was included in the study if she/he 1) was currently, or had been within the past 2 years, on sick leave due to CMDs; 2) had received help from the OHS to return to work; and 3) had experienced that having both work- and home-related demands affected their sick leave or RTW. Moreover, participants were required to speak and understand Swedish due to the focus group design. Exclusion criteria were being a victim of bullying in the workplace or having an initial sick leave episode that exceeded 3 months. These criteria were applied since they were assumed to significantly affect the experiences of how home- and work-related demands affect RTW as well as the experiences of interventions and their effects, the latter which will be explored in a forthcoming paper. Bullying was considered a specific case within the workplace that merits investigation and interventions that are specifically designed to address its particular dynamic in order to facilitate RTW [27]. Also, as this interview study is tied to the main cohort (see above), we aimed to apply the same exclusion and inclusion criteria for all data collections. For the focus group interviews, purposive sampling was applied, and data was collected in two parts.

**Fig. 1:**
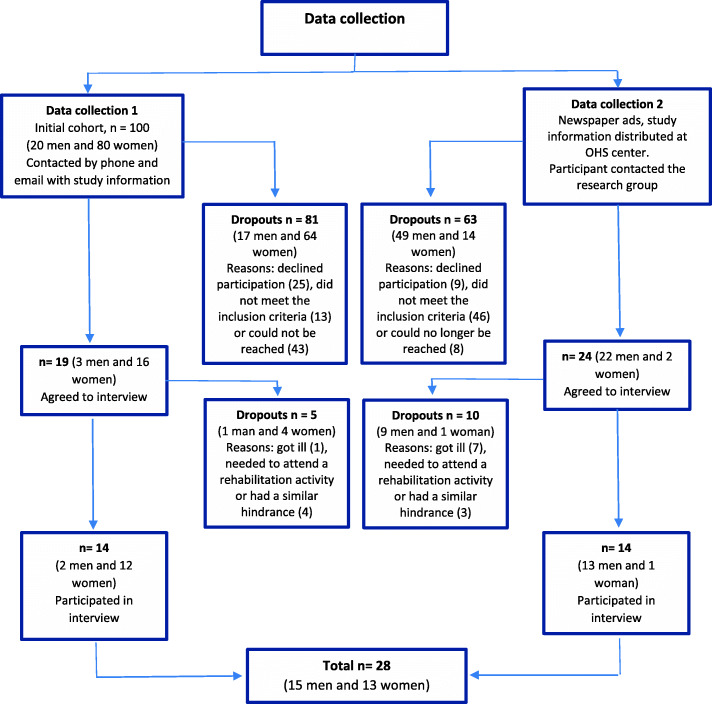
Flow chart of the data collection

First, all participants that at the time had been included in the original cohort (*n* = 100 in April 2017) were contacted by email with information about the study (e.g. study aims and what participation would entail). The email was followed-up by a phone call from the first author (LN), except when an employee had responded to the e-mail and declined participation. A center that had psychologists with expertise in bullying was contacted before the recruitment, and those who had experienced bullying were provided with referral information to the center. No economic incentives for participation were offered. Of the initial cohort, 81 employees declined participation, did not meet the inclusion criteria or could not be reached. Although reasons for declining were not asked due to ethical reasons, some participants mentioned that they were too tired or did not have time to attend an interview. In all, 14 participants attended focus group interviews during the first part of the data collection (Fig. 1).

In order to arrive at data saturation and to increase the number of male participants, a second recruitment was initiated by placing a newspaper advertisement and distributing information about the study at an OHS center. Interested participants then contacted the first author (LN) by phone or email and were provided with more information. Those wishing to participate were screened for inclusion and exclusion criteria and a short phone interview was conducted with questions related to their background and health. The second data collection resulted in 14 additional participants (Fig. 1).

Data saturation was considered complete after considerations of time constraints, the amount of data and data variation. One pilot interview (*n* = 2) and six additional focus groups (*n* = 3–6, a total of 28 participants) were conducted. Two groups had a mixed sex-composition, two groups had only women and two groups had only men. This arrangement of the focus group interviews was deliberately chosen to add to the breadth and variation of the material, as it was assumed that the sex-composition of the group may influence the interactions and discussions among the participants in a way that diversifies how they discuss their experiences. Some differences between the groups according to their sex-composition were noticed, despite the same interview guide being applied in all groups. For example, while the all-female groups spent more time discussing home-related demands and relations to other people, the all-male groups tended to spend more time discussing work-related aspects. Furthermore, in the sex-mixed groups, members of one sex introduced topics to members of the other sex that the latter might not have considered as it had not been relevant to their experiences. Such observations informed subsequent analysis of the data. The interview guide was tested during the pilot interview and the participants’ feedback was asked for with regards to the wording and relevance of the questions. As no changes were made and the discussion among the participants was considered rich and interesting, the pilot interview was included in the analysis. The interviews took place at an OHS and a research institute in two different cities in Sweden.

Information about participant characteristics was gathered in two ways. First, participants of the initial cohort had answered a questionnaire with regards to personal and occupational background as well as mental health. For those who were recruited in the second phase, the corresponding information was gathered via phone interviews prior to the interview date. Second, items pertaining to work and household responsibilities as well as if the participants were currently on sick leave were gathered through short questionnaires from all participants upon arrival at the interviews. This information was gathered to achieve an overview of the study sample as it related to selected characteristics that may influence the focus group discussions. The participants were of various ages and educational backgrounds. Several of the women worked within health care, whereas men mostly worked within engineering, IT and other occupations. It was more common among the participants to have depression, anxiety and/or exhaustion than to not have these conditions, as assessed by the Hospital Anxiety and Depression Scale [28] and the Self-rated Exhaustion Disorder instrument [29]. It was more common among men than among women to answer that home/family affected work negatively. Furthermore, it was more common among women than among men to answer that they were primarily responsible for household work at home. For further participant characteristics, please refer to Table 1.

**Table 1:** Participant characteristics

	Women (***N*** = 13)	Men (***N*** = 15)
	N (%)	N (%)
**Age**		
18–29	2 (15,4)	0 (0,0)
30–39	1 (7,7)	1 (6,7)
40–49	5 (38,5)	5 (33,3)
50–59	3 (23,1)	7 (46,7)
60 -	2 (15,4)	2 (13,3)
**Occupation**		
Health care	7 (53,8)	2 (13,3)
Engineering/IT	1 (7,7)	6 (40,0)
Managers/officials	2 (15,4)	2 (13,3)
Other	3 (23,1)	5 (33,3)
**Living conditions**^a^		
Alone	5 (38,5)	9 (60,0)
Lives with:		
Partner	5 (38,5)	1 (6,7)
Husband/wife	1 (7,7)	3 (20,0)
Children	4 (30,8)	9 (60,0)
Other person	1 (7,7)	0 (0,0)
**Do you have children under the age of 17 living at home**		
No	9 (69,2)	7 (46,7)
Yes	4 (30,8)	8 (53,3)
**Highest completed education**		
Primary school or equivalent	1 (7,7)	2 (13,3)
High school / vocational school	4 (30,8)	5 (33,3)
University / college education /higher academic degree	8 (61,5)	8 (53,3)
**How long have you been working at your current workplace?**		
Less than one year	0 (0,0)	1 (6,7)
1–5 years	4 (30,8)	5 (33,3)
6–10 years	3 (23,1)	7 (46,7)
More than 10 years	6 (46,2)	2 (13,3)
**How many days in the last 12 months have you altogether been away from work due to your own illness (sick leave, care, treatment or examination)?**		
1–7 days	0 (0,0)	0 (0,0)
8–24 days	5 (38,5)	4 (26,7)
25–99 days	3 (23,1)	5 (33,3)
100–365 days	5 (38,5)	6 (40,0)
**Sick-listed at the time of the interview**		
No	8 (61,5)	10 (66,7)
Yes	5 (38,5)	5 (33,3)
**Hospital Anxiety and Depression Scales**		
**Depression**		
No depression	6 (46,2)	3 (20,0)
Mild depression	1 (7,7)	4 (26,7)
Depression	6 (46,2)	8 (53,3)
**Anxiety**		
No anxiety	2 (15,4)	5 (33,3)
Mild anxiety	6 (46,2)	4 (26,7)
Anxiety	5 (38,5)	6 (40,0)
**Self-rated Exhaustion Disorder (s-ED)**		
No s-ED	2 (15,4)	7 (46,7)
Mild s-ED	2 (15,4)	3 (20,0)
S-ED	9 (69,2)	5 (33,3)
**Who is primarily responsible for the household work at home?**		
Myself	9 (69,2)	4 (26,7)
Someone else	0 (0)	0 (0)
We divide equally	4 (30,8)	11 (73,3)
**Does your work affect your home and family life in a negative way?**^b^		
Very rarely or never	0 (0)	1 (6,7)
Rarely	0 (0)	1 (6,7)
Occasionally	5 (38,5)	7 (46,7)
Quite often	5 (38,5)	4 (26,7)
Very often or always	2 (15,4)	2 (13,3)
Mean value (SD)	3,7 (0,8)	3,3 (1,0)
**Does your home / family affect your work in a negative way?**^**b**^		
Very rarely or never	3 (23,1)	3 (20,0)
Rarely	4 (30,8)	2 (13,3)
Occasionally	5 (38,5)	5 (33,3)
Quite often	0 (0)	4 (26,7)
Very often or always	0 (0)	1 (6,7)
Mean value (SD)	2,2 (0,8)	2,9 (1,2)

^a^Participants were allowed to choose multiple responses on this question. Some have chosen both “alone” and “living with children”, which might reflect that some lived every other week alone, and every other week with their children

^b^ One of the female respondents answered both “rarely” and “occasionally”, and the response was hence excluded

### Ethical considerations

Ethical approval for the main study [26] (registration number 2015/549–31/1) as well as for its current qualitative part (registration number 2016/972–32 and 2017/2021–32) has been granted by the Regional Ethical Review Board in Stockholm. Amongst other things, it was emphasized that study participation is voluntary and that the participants could withdraw from the study at all stages of the research and without giving a reason. Informed, written consent for study participation and for the interviews to be audio-recorded was obtained from all participants during each interview session.

### Interviews

A reminder e-mail and/or text message was sent to all participants 1 week prior to the interview date. Upon arriving to the interview, participants were asked to fill the survey with five questions related to their work and household responsibilities as well as if they were currently on sick leave (see Table 1). Coffee and snacks were provided, allowing for an informal chat before the commencement of the interview. A moderator (TH in the first interview and LN in the remaining ones) and a co-moderator (LN in the first interview and TH in the remaining ones) participated in all interviews. Both are female researchers and experienced interviewers with doctoral degrees. TH is also a registered occupational therapist. Their academic backgrounds and research interests were presented to the participants. The moderator explained why a focus group method was chosen, encouraged the participants to address each other and ensured that everyone took part in the discussion. The interview guide included areas that corresponded to the aims of the current and the forthcoming (see above) study (see Supplementary File 1). The areas were (in the order in which they appeared): “Home- and work-related demands and how they affect return to work”, “Experiences of interventions and their effects” and “Have the experiences been affected by being a man or a woman”. The first area corresponds to the aim of the current study, while the two following areas are explored in forthcoming manuscripts. The area of the current study “Home- and work-related demands and how they affect return to work” included the following questions (in the order of appearance): how the participants would describe home- and work-related demands before their sick leave period, with a follow-up question of how it affected their well-being; the home- and work-related demands that were experienced as most difficult to handle; how the demands changed during the sick leave period; how the demands influenced their return to work with two follow-up questions: which home- and work-related demands were the largest obstacles for returning to work and which work- and home-related resources were supportive for returning to work. The questions in the guide reflect the understanding of RTW as a process and the guide hence includes questions on the time before, during and after the sickness absence period to help participants contextualize the RTW. During the analysis, data that related to the experiences during and after the sick leave period were focused on. The areas were followed by questions such as “how did you experience that the demands affected your RTW?” and probes such as “Can you tell more about…” and “Interesting, any other opinions on this?” were used [30]. The guide also included a question on what demands they felt was the main reason for their sick leave period. The interview lengths varied between an hour and half to two hours and were carried out between June 2017 and March 2018. Field notes were not applied. All interviews were audio-recorded and subsequently transcribed verbatim and pseudonymised by a professional transcription company.

### Data analysis

Data analysis was initiated once all interviews were completed. Conventional content analysis was chosen, which is appropriate when there is little or fragmented previous knowledge on the subject [30]. According to Elo & Kyngäs [31], preparation, organizing and reporting are the three main phases of a content analysis. In the preparation phase of the data, the interview tapes were listened to. Next, the interview transcripts consisting of 192 single-spaced pages were read to gain a sense of the whole and to become immersed in the data. For organizing the data, an inductive approach was applied. This includes open coding, creating categories and an abstraction phase [31]. We began our open coding phase of the analysis by highlighting the parts that related to the study aim [31]. The interview transcripts were re-read and headings as well as notes were written into the margins until no further aspects could be thought of. Finally, these were transferred to coding sheets and several preliminary categories were thereafter freely derived. In order to create categories, the preliminary categories were compared with one another and grouped so that those that “belonged” to one another were joined [31]. The open coding and creating categories phases were done by TH and LN for the first interview and by LN only for the rest of the data. Including two researchers throughout the whole process for the first interview was done so that the emerging categories could be tested, challenged and deepened in order to improve credibility of the analysis. Furthermore, TH received all the open coding sheets and read the emerging, preliminary categories created under the second phase of the analysis to scrutinize and discuss them, which sometimes led to changes in how the categories were created. Emerging patterns of the women’s and men’s experiences werescrutinized, compared and written down in reflective notes during all steps of the analysis. Finally, in the abstraction phase, the preliminary categories were considered in relation to each other and those considered to belong to the same category were merged and sub-categories were developed. Examples of content characteristic words that were used to create categories are “internal demands” and “overbearing feeling of responsibility”. The final abstraction phase was considered complete when the categories were deemed stable, coherent and distinct from one another [31]. The categories and sub-categories in the abstraction phase were discussed regularly among all authors to achieve accord of the interpretation. GB is a male professor in occupational health science with basic education in psychology and sociology. Alternative interpretations were considered. Feedback of transcripts or findings from the study participants was not applied.

## Results

The findings depict work- and home-related demands and resources that influenced the participants’ RTW. While the study aim was to explore aspects of work and home, additional considerations related to internal demands and involved actors were also found (see Table 2 for all categories and sub-categories). Women spent more time discussing demands related to household work and the well-being of others in relation to RTW, whereas men spoke more of the impact that structural work environment problems had on RTW. Furthermore, women spoke extensively of the emotional work involved in care-taking professions, whereas men spoke more about unwritten norms of being a “hard worker”. Both women and men experienced internal demands, but in partly different ways.

**Table 2:** Overview of the results. Main and sub-categories

Home-related demands and resources^a^	
*Not on sick leave for home-related demands* ^a^ • E.g. women felt responsible for their homes, feelings of guilt; men described fewer obstacles to lowering demands on household work, less feelings of guilt *Feeling responsible for relationships and the well-being of others*^a^ • E.g. women took care of others and managed a web of relationships; men felt supported in taking care of social relations to support RTW *An affected economy*^a^ • Economically strenuous circumstances evoked feelings of shame and regret among men; women did not mention such aspects *Finding energizing activities and creating routines* ^a^ • Women were helped by close friends who provided valuable support in initiating outings; men did not mention such aspects	
Work-related demands and resources^a^	
*Encountering tough emotions and an over-bearing feeling of responsibility at work*^a^ • E.g. women within the social and health care sectors questioned their capabilities in dealing with the responsibility of their clients and the tough emotional demands when back at work; a few men worked within this sector and did not discuss such aspects as extensively *Continued work-related demands create un-certainty about the future*^a^ • Women felt that they were the main problem; men held managers responsible but felt resigned when no change happened *Loss of boundaries* ^a^ • Men described aspects of being at loss of boundaries at work *(Desired) support from managers and colleagues*	
Internal demands and resources^a^ • E.g. women blamed themselves and doubted their capabilities; men felt they failed to live up to demands of a “strong worker”, feelings of shame	
Demands and resources linked to involved actors	

^a^Differences emerged between women and men, with examples in bullet points

### Home-related demands and resources

#### Not on sick leave for home-related demands

Although most participants felt that job-related demands had been the primary reason for their sick leave, home-related demands also influenced their experiences. Female participants spoke at length of how their partners expected them to take care of household demands during their newly gained “free time” as sick-listed. The household demands included taking care of children, cooking, cleaning and managing timetables, administrative tasks as well as general planning for the family. The women felt primarily responsible for their homes and described themselves as project leaders. This responsibility deterred many from reducing these tasks in favor of rest and recuperation during their RTW, especially for those with children at home (female participant 1) but in some cases also for those without (female participant 2):

*Female participant 1: “During that period, well... [we] had real difficulties dealing with the situation that occurred at home, because ‘mom was home’[.]So my partner did very little household work during this period. I said [to him] ‘but you will also have to… I’m not at home with free time [on my hands], I’m just supposed to take it easy and take care of my health.’”*

*[…]*

*Female participant 2: “I agree with this, I also felt responsible, like ‘I am at home, so I should be able to do the laundry, take out the dishes, and take care of the cleaning…’ I used to do this before and I should be able to do it now[.]”* (Women, focus group 3, sex-mixed group.)

Consequently, the women tried to lower the demands placed on them in relation to household work to facilitate their RTW. However, this could be difficult not only for their partners to accept, but also for the women themselves. The women recounted how difficult it could be to just relax when their partners cleaned, without joining in the work. However, those who had help from a psychologist in informing their partners that they were not able to perform household choirs during their sick leave period and instead needed the time to rest, experienced this as a valuable facilitator during RTW. Also, those who had not access to such facilitating factors expressed a need for them to help ease the burden of home-related demands. One woman recounts how valuable it was when her partner came to an appointment where a psychologist had explained what mental ill health is and why it was important that she rest and do less household work: *“It’s like it almost has set the agenda for the rest of my rehabilitation process.”* (Woman, focus group 3, sex-mixed group.) While lowering household demands was essential to their RTW, the feeling of being understood could in itself relieve part of the experienced burden.

There was a larger variation of experiences related to household demands among the male participants. Although it was to a lesser extent, men also spoke of how the household demands influenced their RTW, and how they tried to lower these demands. Although referring to a smaller number of men, those who came or whose partners came from other countries, and young men wanted to be active fathers, spend time with their children and take care of household tasks, which created difficulties in finding a good work-home balance upon RTW. For example, referring to another participant who had mentioned similar aspects, a participant with a spouse from a country outside of the Nordic countries with whom he had children said: “*I feel that I might have had some more problems, if you think about my wife who isn’t integrated in the society and so on[.] And I assume that you [another participant] have had similar problems[.] You know, one has to explain [how to pay a bill or where to buy things]. And that made me feel like I can’t hand such things over to my wife.*” (Man, focus group 4, all-male group.) However, the men did not describe obstacles to lowering demands on household work during RTW, or feelings of guilt to the same extent as the women: rather the men expressed a more naturally occurring emotional and practical support from their partners. Generally, their partners would quickly accept how they felt and take over cooking, cleaning, as well as childcare and were a considerable resource for them, enabling rest and recuperation in support of their RTW. For example, several men visited friends or took a vacation to a warmer country while their partners handled household demands and childcare: “*That’s where I felt the best [at a warm country]. Then… I didn’t have the energy to spend time with my family.”* (Man, focus group 4, all-male group.) The female participants did not seem to have or think of similar resources.

#### Feeling responsible for relationships and the well-being of others

Women spoke extensively of the demands they felt for the relationships around them, which caused additional strain on their efforts to recover and return to work. These demands were subtly interwoven into their everyday lives and were hence also more difficult to reduce in favor of rest. They took care of family members in distress, tuned in on their emotions, provided reassurance and gave other emotional support. They also painted a picture of being the spider in a web of relationships, for example by organizing gatherings for relatives or friends, caring for their partner’s relationships or being the go-to person when someone needed help. They became aware of this during the sick leave period and wanted to tread out of these responsibilities to reduce the amount of demands. This was difficult, however, as they had been in a care-taking role for a long time, as in the case of these women who also had children at home:

*Female 1: “It’s also like you enter a role, that you may have had early in life, and suddenly you realize that the role has grown much bigger and… you can’t just back down from it. I can also recognize that aspect [mentioned by a previous female participant] that you become like a federal relationship center of the family and… and when you begin to realize that yourself and then try to tread out of it, it takes time.*”

*[…]*

*Female 2: “One has created a role, as you say. […] And life doesn’t revolve around me, but I’m holding onto all the threads. And to tread out of that role is […] difficult[.] When you try to let go of something that you have built during all these years, with all the roles that you have towards work and relatives and friends and family and everything.”* (Women, focus group 2, all-female group.)

The above quote also echoes how the general caretaking demands mainly experienced by female participants were reinforced by feeling responsible for the well-being of others, at home and also in care-taking professions, where the women felt responsible for the well-being of their clients. At times, colleagues were also spoken of as people who were close to the participants, and particularly female participants often felt guilty and experienced additional strain during their RTW as they saw how stressed their colleagues were, and yet they could not participate with full strength to help their overworked colleagues.

Men, particularly those of younger generations, from other cultural backgrounds or whose partners came from other countries or had suddenly become ill, could also discuss feeling responsible for others and the hindering effects this had on their RTW. However, in most cases it was common for men to express how their wives took care of social relations during their sick leave in a way that supported their RTW, as recounted by a male participant who also had children at home: “*And then she took care of... all the planning and so on, because it was quite demanding, all this with birthdays and we need to go there, visiting sisters, or whatever it is, so… I didn’t meddle in any of that. I just sat next to her in the car and tagged along*.” (Man, pilot interview, sex-mixed group.)

#### An affected household economy

For most participants, the sick leave period entailed a weakened household economy that enhanced feelings of stress and anxiety. For some, the economic demands were cited as the main reason for returning to work, even in cases where they did not feel well enough to do so. As one male participant with a vocational training expressed: “*Now I’m working 50 %, and that’s something I’m mostly doing because of the economic… economically speaking it doesn’t work out to be sick-listed 100 %. So I’m forced to return to work, simply enough*…” (Man, focus group 4, all-male group.) The economic frailty continued also when back at work, leaving participants worried if they could manage a new sick leave period, were it to happen again. For example, one participant took a loan before returning to work to secure the option of quitting her job if need be. Furthermore, the economically strenuous circumstances evoked feelings of shame and regret among the male participants, who often felt economically responsible for the family.

In a minority of cases, however, the participants considered their economic situation more of a resource for their RTW. For example, one participant was initially too tired to apply for sickness benefit and could wait a few months until he begun the application process as he had the economic means to do so.

#### Finding energizing activities and creating routines

After an initial period of rest and as the sick leave progressed, both women and men began to feel the need for something active to do so that they wouldn’t remain passive for too long. Many resumed activities they had been doing before the sick-listing such as participating in a voluntary group or riding a motorcycle, which was described both among women and men. Alternatively, they began new activities such as going for long walks. In addition, women also spoke of being helped by close friends who would initiate outings of different sorts, as expressed by a female participant who lived alone: “*I had friends who took me to things, and then I didn’t need to plan anything, but I have just been able to tag along. That’s been so nice; otherwise I wouldn’t have done it, if I had needed to be in charge of it.”* (Woman, focus group 2, all-female group.) While the female participants generally needed to withdraw from social interactions during and after the sick leave, close relationships that were free of demands provided valuable support. Furthermore, some home-related demands, such as driving one’s partner to work or children to daycare, provided a good daily structure and took their minds off worries.

### Work-related demands and resources

#### Encountering tough emotions and an overbearing feeling of responsibility at work

Upon RTW, both women and men in care-taking jobs strived to lower demands related to tough emotions at work and yet be capable of doing a good job. As they often encountered people in vulnerable situations, it was a challenge to feel concern and engagement towards their clients, but not burdened by their plight. The participants doubted their capabilities to handle tough emotions that they encountered at work including anger, resentment and despair expressed by clients and their families, and to respond to these situations with the appropriate kind of reactions (e.g. not becoming angry themselves). They strived to follow advice from their managers who told them to let things roll off like the water off a duck’s back, but this was easier said than done, especially among the women in care-taking professions:

*Female participant 1:” Water off a duck’s back, water off a duck’s back, given how much we have to take, and the responsibility… did we make the correct assessments, have you written it in the journal, have you made a police enquiry, have you made an assessment regarding… compulsory care. Such things begin to spin in your head. But I think I need to learn that part[.]*

*Female participant 2: It really is… it’s a fine line, this water off a duck’s back, water off a duck’s back, to have this protection[.] Because at the same time, we work with people, […] you have to enter the role with empathy.”* (Women, focus group 3, sex-mixed group.)

Many women in care-taking professions questioned if they could continue working in their jobs in the long-run and felt that this depended on their capabilities of dealing with the responsibility of their clients and the tough emotional demands imposed by their work.

While this aspect was mostly discussed by women, the few men that also worked in care-taking professions mentioned similar concerns. One of them had troubles finding new assignments at his job as he no longer could bear to work with vulnerable youth and all assignments had some involvement with such issues. The other also expressed that he needed to learn to let things roll off like water off a duck’s back; however, in contrast to the women, he had found this easy to accomplish during his RTW.

#### Continued work-related demands create uncertainty about the future

The participants spoke extensively of issues and concerns that they met at their workplace upon their return. Many met the same, unresolved issues as before sick-listing. For example, work schedules were demanding and poorly planned, the amount of work was unreasonable, or conflicts with the manager pertained. In addition, some were met with a new set of demands: divisions merged, or large-scale policies changed. Consequently, many doubted how long they would be able to continue working at their workplace, as they had to deal with both old and new demands in combination with a lowered work capacity. Men often spoke of beginning to stand up against unreasonable work demands or to decline added assignments. Having returned to work was accompanied by feelings of anxiety and insecurity regarding one’s future work capabilities, conditions and possibilities. Women often felt that they were the main problem and that they needed to choose the job they had or a new line of work, as described by a female participant working within the care-sector: *“I feel like I’ve had to fix a lot myself, and fix myself. Okay, should I accept the job as it is and all that it comes with, or should I do something else? And I didn’t want that, so then I have to accept it as it is, and then try to find strategies myself.”* (Woman, focus group 3, sex-mixed group.) Men, on the other hand, more often distributed responsibility to poor work environments and demanded more of their managers, as expressed by a male participant within the IT sector: *“There is extreme rotation, there are a lot of people who are on sick leave, even at HR. We have addressed it with the management, but no one wants to take responsibility there.”* (Man, focus group 6, all-male group.)

In addition, male participants often described a process of resignation as a way to deal with the continued work-related demands upon their return. They gave suggestions to their managers on how work-related conditions could be improved, but even when managers were sympathetic toward the suggestions, there were no resources or possibilities to implement them. Consequently, the male participants felt the need to surrender to the poor working conditions and to doing their jobs inadequately in order to continue working where they were. However, they were reluctant to do so and felt contempt as well as frustration that they were left with no further options. The process of resignation misplaced structural problems on the individuals, hindering sustainable working conditions for the long run.

#### Loss of boundaries

The male interviewees, particularly, described different aspects of being at loss of boundaries at work. While this peaked right before their sick leave, they also spent a lot of time thinking about these issues upon their RTW. Not knowing what was “good enough” in combination with high demands that the participants put on themselves led to limitless work where they pushed themselves beyond their capacities. Not knowing what was good enough was also spoken of among the women, although to a lesser extent. Men described how unwritten norms of “hard work” created borderless work, facilitated by laptops and work phones that extended work to their homes and weekends. This became challenging to relate to during RTW as they had a lowered capacity, creating insecurity about if and when they were to rest. Once participants returned to work, they continued to feel confused about how much the employers in fact expected them to work. Creating space for rest became hence an important resource for managing such demands, although the participants were frightened to notice how hard it was to recharge their batteries. They needed to be vigilant at all times and worried about ending up on sick leave anew:

*Male person 1: “But I feel a bit like a sober alcoholic, [...] if you keep on stressing, and start spinning faster and faster, as you did before, you will soon be there again[.]”*

*Male person 2: “Yes, I also think that’s difficult to know, when am I well? [...]”*

*Male person 3: “Yes, exactly.”* (Men, focus group 4, all-male group.)

They could take several micropauses per day when they felt the need or follow scheduled pauses to take their minds of job-related tasks. In addition, many made intentional efforts to draw clearer boundaries between themselves and their work by asking superiors to clarify their expectations of them or by leaving work phones at work, which instilled a renewed sense of confidence in their capabilities to manage their work.

#### (Desired) support from managers and colleagues

Managers’ and colleagues’ support or lack thereof played a central role for the participants’ RTW, which was described in similar ways by women and men. Those who described supporting managers and colleagues felt that they were seen, accepted and understood. Supportive managers adapted workloads, assignments and working hours in accordance with the employee’s needs. They expressed trust in the employee’s abilities, helped them lower their demands on themselves, stopped them from working too much, maintained a respectful attitude and generally had their best interest in mind as expressed by a participant in the care-sector: *“But the boss that I have now, she is very wise, and she stops me ‘You understand [name of the participant], you can’t [work like that], instead we will now do like this’. Because I will spiral out of control[.]* (Woman, focus group 2, all-female group.)

Concerned and engaged colleagues who asked how it felt to be back were also described as important resources that enabled the participants to return to work with their head held high. Resources related to managerial support made the threshold for RTW feel lower.

However, many felt alone and unsupported. Some managers blamed them for having worked too much and for bringing the situation upon themselves, which created anger, shame and self-doubt. Many wished for greater sensitivity among managers towards their needs, as well as support in communicating these needs to their colleagues, as described by a participant who worked in a private company:

“*I’ve felt that my boss hasn’t taken this with… the other [colleagues] and talked about, like, ‘[name of participant] is feeling like this and needing that, think about such and such things when sending emails so it won’t cause stress’. But I haven’t gotten that help either, because nobody really knows [what I need]*.” (Woman, focus group 1, all-female group.)

Instead, managers and colleagues often assumed what the participants needed or wanted. Lack of managerial help in adjusting work-related aspects, lack of trust and lack of understanding for the employees altered capabilities created stress and concern regarding the sustainability of their RTW.

### Internal demands and resources

While the participants experienced high demands both at work and at home, they also felt that they could influence these by adjusting the demands that they placed on themselves. Participants described how they before the sick leave period placed high demands on themselves and wanted to give everything at all times both at work and at home, or else they felt bad. Such internal demands could hinder them from declining high demands placed on them by others and was mentioned by many as the most difficult obstacle during RTW. Hence, demanding less of themselves became an important resource for a successful RTW. However, even though their bodies could not be pushed to do more, lowering demands on one’s self was difficult to accomplish:

*Male participant 1: “My body and mind want two completely separate things[.] When I got back to work, when I began working at another workplace, then ... I know what I can deliver and what I want to deliver. But the body [...] said no*.”

*[...]*

*Male participant 2: “I can recognize this so well also, that the mind… that the psyche wants one thing and the body something completely different. And the body is protesting wildly over something that the psyche wants it to do.”* (Men, focus group 5, sex-mixed group.)

Reducing high internal demands was described as a continuous process where they regularly needed to check in with their bodies’ signals. This was also directly applied to their sick leave process, where many tried to be the “good patient” who met others’ expectations of the recovery process, also when it didn’t mirror their own needs. This led to some returning to work quicker than they were capable of, which resulted in new and longer sick leave spells. Participants then begun to reduce such internal demands by tuning into their own needs and matching their own demands to their capabilities.

Particularly, men felt that being on sick leave meant they had failed to live up to demands of being a “strong worker”. This often led to feelings of shame, which needed to be worked through before they could return to work with a sense of self-respect, as for a male participant who worked within a private company:” *Right after I crashed, I felt such shame… It was so shameful, and… and wrong and… I mean, it didn’t go at all with my image of myself.”* However, after beginning to speak openly about being on sick leave, the same participant:” *felt much better then, every time [that he spoke of the sick leave to someone] and finally I didn’t really feel bad about it at all*.” (Man, pilot interview, sex-mixed group.) For women, on the other hand, the high internal demands were reflected in a tendency to blame themselves for their situation and to feel as though they were the main problem, even when they were dealing with highly burdensome working environments:

*“I go home, and I feel like a problem the whole day. After work. And I don’t have the strength. And I’m just feeling, what am I doing wrong, what could I have done differently for this to have turned out differently? But I realize that it doesn’t matter how much I take on myself. How much CBT [cognitive behavioral therapy] and therapy that I commit to, if no one else will change.”* (Woman, focus group 1, all-female group.)

Hence, they often doubted their capabilities and concluded that they either needed to change their line of work or accept the conditions as they are. However, when they began to demand less of themselves during their RTW, some also felt less responsible for their situation. This shift in itself could alleviate the experienced demands, as conveyed by the female participant above.

### Demands and resources linked to involved actors

The participants spoke of new, bureaucratic-related demands that needed their attention to keep the sick leave period going. During a time when they wanted to rest and be passive, new “sick leave choirs” from involved actors required them to be active and alert.

In particular, the Swedish Social Insurance Agency was portrayed as an impediment in the sick leave process. Interactions with the agency were often described as unpredictable, rigid and lacking in transparency. Decisions felt erratic and hard to understand, and regulations were complex. A frequent depiction was how the doctor’s certificate or an RTW plan was rejected by the agency without apparent reason. Participants, together with their doctors, psychologists and employers, were left wondering how the agency could have better insight into their situation than someone who had met them and spent time mapping their needs. Many felt the need to be “*street smart*”, as one male participant expressed it, and that “*one would maybe need a course on the Social Insurance Agency. At least a crash course. I did get a lot of information… from my doctor, in fact, about what to do and how, but… but there were many things that I didn’t get.*” (Man, pilot interview 6, sex-mixed group.) Other strenuous aspects included when officials were replaced and processes needed to start anew, lacking guidance regarding rules and regulations, not understanding why certain information was needed, having difficulties getting hold of their contacts, or receiving demands that didn’t fit their particular situations. At times, the agency made mistakes that needed to be corrected, but it could be difficult to get hold of an official and schedules were not adapted to the needs of the sick-listed individual. Participants felt that the representatives talked quickly and unkindly, which made the information that was being relayed to them more difficult to understand. They stated that being in contact with the agency was a hindrance to their recovery.

Also in relation to other actors, participants felt the need to become immersed and knowledgeable in the world of authorities during their sick leave period. They learnt about rules and regulations, chased doctors, officials, statements and referrals, answered multiple questions from several places, filled and sent in documents and had frequent appointments. They conveyed information between parties such as the primary health care, the OHS, the National Social Insurance Agency and the workplace, as well as between different professions within these. Simultaneously, they were themselves confused about the role and needs of each enactor and didn’t know how to muster the required energy to tackle all demands. They made efforts to keep track of a large number of threads, which required cognitive functions that were impaired among the participants. Some described how contacts with the authorities deepened their exhaustion and most felt that it was a hindrance to RTW. The participants expressed a need for more concrete help with the bureaucratic demands. They suggested a timeline and systematic overview of tasks that needed to be accomplished, the order in which they needed to be completed and what agency to contact for each task. Concrete suggestions included a checklist, a pamphlet and/or a personal coordinator: *“It is not so easy to know where to turn to with everything. Where to call, which place to contact, who can help you and so on[.] You got to be damned healthy to be sick, in many ways. If one could only get a list [.] Tips and advice.”* (Woman, focus group 2, all-female group.)

However, some participants had very positive experiences of their contacts with the agency representative, who was a resource for their return to work. In these instances, the representatives were described as flexible, respectful, clear and as genuinely concerned about their situation. Similar interactions were described as a large help in their recovery processes.

## Discussion

Although CMD-related sick leave is prevalent and women have a higher risk for such sick leave than men, few studies have considered how combined work- and home-related demands as well as resources influence RTW, and whether women’s and men’s experiences might differ. The present study considered how work- and home-related demands and resources influence RTW, and to compare how patterns of these are described by women and men sick-listed due to CMDs. In general, a traditional gender structure emerged. Women spoke more of the demands related to household work and the well-being of others in relation to RTW, whereas men spent more time discussing the impact that structural work environment problems had on RTW. Overall, the male and female participants worked in different sectors (see Table 1), which is partly reflected in the differences in work-related demands identified by women compared with men. Women spoke extensively of the emotional work involved in care-taking professions, whereas men spoke more about unwritten norms of being a “hard worker” within sectors such as IT. Both women and men experienced internal demands, but in partly different ways. The findings consist of four main categories and associated sub-categories and are depicted in Table 2. Differences among the women and men were observed for three out of the four main categories.

### The effect of home-related demands on women’s RTW

Whereas home-related demands became pronounced during RTW of women and hindered rest, the domestic arena was generally a place for practical and emotional support for men. This finding is in agreement with that of a qualitative study on domestic strain during RTW of women and men sick-listed for musculoskeletal disorders [23]. The results are also consistent with and point to the sorts of experiences that might underlie the finding of a longitudinal study which found that men seemed to have better balance between employment, domestic work and leisure than women, and that leisure might be a more pronounced health protector for men than for women [32]. The findings may also provide context to interpret the findings of another study that it is not solely the division of domestic work but also how satisfied couples are with that division that affects health [33] and extends this to the context of RTW. It is possible that the RTW presents a period when women become more dissatisfied with the division of labor, as they need more time to rest and recuperate than before. Although traditional gender norms are arguably changing in Sweden (for example, men are taking an increasing amount of parental leave), women still account for the largest share of parental leave and spend the largest amount of time on unpaid work [34]. It is possible that gender inequalities at the structural level situate women and men differently within the RTW process. Our results lend additional weight to previous calls for including home-related aspects in addition to work-related ones for a fuller account of the RTW process [8, 13, 14, 20] and to continue the investigation of possible gender differences among these [12].

### The effect of emotional and care-taking demands on RTW among women and men

Emotional demands at work hindered RTW among the interviewed women within care-taking professions; in part, this may reflect Sweden’s gender segregated labor market [34]. Interviewees provided examples of factors such as high demands and low control that characterize female dominated occupations and that are known to increase the risk of CMD-related sick leave [35, 36]. Furthermore, the interviewees’ experiences were consistent with the “emotional dissonance” that has been documented in care-taking professions – that is, conflicts between the emotions felt and expressed at work, which may increase the risk for burnout and job dissatisfaction [37]. In light of the findings, this may be relevant for studies to pursue further in relation to RTW among employees with CMDs.

The interviewees within care-taking professions tried to strike a balance between creating a certain amount of emotional distance from the patient and expressing genuinely felt concern, conceptualized as “detached concern” elsewhere [37, 38]. Interestingly, striking such a balance was especially difficult for the women. It is possible that this difficulty on part of the women is due to gender norms that advocate a care-taking role for women, with experienced responsibility for the well-being of others [39]. Similar findings have also been reported in another Swedish study [8] and a Dutch study conducted among women [9]. These findings imply that not only the occupation itself, but also the practice of gender norms within a particular occupation may affect RTW. However, our study only included few men within care-taking professions. This warrants undertaking future studies designed to look at this issue in more depth and with equal proportions of women and men within care-taking professions.

Gender norms that impose a care-taking role [39] could also explain why the interviewed women felt responsible not only for the well-being of their family at home but also their colleagues at work. Furthermore, experiencing this both at work and at home reinforced one another, underscoring the need to study both domains and their interplay [8]. The role overload theory posits that multiple roles may have negative effects on sick leave. Although the findings are not conclusive [15], our findings are consistent with the predictions of this theory. By contrast, the present interview-based findings did not support role enhancement theory, which is a counterpart to role overload theory and posits that multiple roles come with benefits that make it easier to manage these roles, and can be positive for one’s health [16].

Some of the men also spoke of care-taking demands, particularly those who had sick spouses, who had children, or who came from outside the Nordic countries or whose partners came from abroad and had difficulties in adjusting to Sweden. While representing a minority theme in the analysis, the experiences of the men who came from outside the Nordic countries or whose partners came from abroad echo the findings of a study that found that supervisors and occupational physicians needed more attention for ethnic minorities in mental health care to prevent a delayed RTW [40]. Immigrants have an increased risk of sickness benefit compared to native Swedes. This has primarily been explained by the fact that immigrants often work in professions with higher rates of sick leave spells. However, even after controlling for socioeconomic background and labor market time, an increased risk for sickness benefit remains among immigrants in Sweden [41]. This group may thus deserve greater attention. Such findings imply that not only gender, but also the relationship of other aspects such as age and ethnicity to the demands and resources during RTW, should be explored. Intersectional theory could inform a suitable approach, because it allows for multiple axes of analysis that account for the effects of different social categories on a person’s experiences [42]. Such an approach could be applied by future studies to deepen and expand on our understanding of the experiences of demands and resources among men from outside the Nordic countries or among men whose partners come from abroad.

### Demands experienced by men during RTW

The interviewed men tended to blame structural-level demands at work for a hampered RTW. This is in contrast with the interviewed women, who tended to blame themselves for the difficulties experienced at work. Just as such experiences affect women’s health negatively [7], the men’s reported experiences also seemed to affect their health and RTW negatively, as they felt contempt and resignation that no changes at the structural level occurred, despite their best efforts. As men may identify strongly with their professional work [43], it would be important to support men in these situations in ways that enhance their well-being and facilitate RTW in the long run.

Previous work has documented masculinity norms related to bearing economic responsibility for the family [44] and being a hard worker [11]. These norms may inhibit men from seeking help [10], especially for mental-health issues that may be seen to be contradicting such norms [11]. Indeed, in the present study men from various socio-economic backgrounds confirmed how they struggled to lower demands on themselves and worked through feelings of shame related to mental ill health while retaining a positive self-image of being a capable worker. One study found that men and women both find it more difficult to identify mental ill health among men, and, furthermore, that men find it especially difficult to do [45]. This highlights the need for the support provided to men during RTW to be sensitive to and account for norms related to masculinity.

### Clinical implications

In conjunction with other rehabilitation activities, the findings point to the need for the OHS practitioners to investigate work-home balance during RTW and to offer scheduling a meeting with the sick-listed employee and his/her partner to oversee possible changes that can be made in home-related demands. Such a meeting could also provide an opportunity, particularly for sick-listed women, to renegotiate the gender distribution of childcare and home-related demands in a way that supports a sustainable RTW also in the long-run. This provides an example of how to support employees with CMDs in such ways that they will continue to stay and thrive at work also in the long run, which has been suggested in a recent paper [14].

The women (and some men) considered their friends a significant resource during RTW, a finding that is in line with a previous study on how social capital outside work has been found to increase the work ability and facilitate RTW among women [46]. This points to the need for OHS professionals to map resources both at work and at home to enable RTW. Furthermore, employees from abroad and employees with partners from abroad may need particular attention, because of the added demands imposed by the lack of close networks of friends and/or family nearby.

Finally, the interviewed women as well as men expressed the need for a checklist, pamphlet and/or personal coordinator to help gain an overview of the demands and additional tasks required by the actors involved in the RTW process. In Sweden, there is increasing effort to implement a rehabilitation coordinator within primary care to facilitate, for example, coordination of administrative tasks and communication between involved parties [47]. This seems to correspond to the needs expressed by the participants and could also be extended to the OHS setting. Furthermore, a checklist for OHS professionals may be useful; it could be distributed to patients with CMDs to take home and refer to during the different stages of their RTW. The checklist could be used as support for the sick-listed employee in the communication towards other involved actors in the rehabilitation process.

### Methodological considerations

A majority from the initial cohort could not be reached or declined participation in the interview study. Often cited reasons for declining were being too tired or not finding the time to participate. This may implicate that the study results do not include experiences of those who were most tired or had the least amount of available time in their everyday lives. Individual interviews via phone could possibly be considered by future studies to reach this population. Furthermore, the focus groups included both same-sex and different-sex participant compositions. This arrangement may have added to the breadth and variation of the material, as the different sex-compositions may influence participant interactions in a way that diversifies how they discuss their experiences. A possible disadvantage is that in more homogenous sex-compositions certain themes may have grown stronger. Except for the first interview, only one researcher (LN) conducted the open coding phase of the data analysis. Having two researchers throughout the open coding process would have strengthened the validity of this phase. However, this was not possible due to time constraints. Instead, TH received all the open coding sheets and all categories were scrutinized by TH during their creation. Furthermore, during the coding process in this manuscript the authors were in agreement about most of the coding and when disagreements arose, they were carefully discussed, and data was considered in light of the aim until consensus was reached between LN and TH. Also, all authors discussed all categories during the abstraction phase of the analysis. Interrater reliability was not calculated. In addition, the results mainly focus on demands, which reflects that the questions in the interview guide focused on demands for the period before and during the sick leave period, before inquiring about demands and resources when returning to work. Finally, a majority of female participants was working within health care, whereas the most common workplace for participating men was within engineering/IT. In addition, the study sample consists of employees with CMDs who experience that home- and work-related demands affect their RTW and who have received help from the OHS to return to work. This implies that the study results are valid for this sample. Participants working in other fields, for example, would possibly have other experiences of particularly the work-related demands and resources. Furthermore, that women and men were partly in different occupations also needs to be considered when comparing the patterns of experiences. The gender differences that are identified in the categories related to work-related resources and demands are likely to be influenced by the occupation in which they are experienced. Hence, it would be an interesting area for future studies to look at women and men in both male and in female dominated occupations to study the presumably parallel influences of both gender norms and occupational settings, as indicated by some of the results in the current manuscript with regards to care-taking occupations.

Six focus groups and one pilot interview were included: although the sizes and amounts of focus groups vary greatly between studies [48], this adheres to a common rule of thumb of having between four to six focus groups [49]. All but the pilot interview and one of our focus groups (with three participants) achieved a size of between four and eight participants, which may be considered ideal [25]. The smaller number in one group resulted from cancellations that occurred in close conjunction with the scheduled interviews. Across all groups, the discussions were rich and lively, which indicates that the group sizes were conducive to fruitful interactions among the participants. Moreover, smaller groups are easier to manage [50] and are preferable when the participants are particularly involved in or affected by the topic at hand [49], as in the present study.

## Conclusion

Home-related demands and resources influence RTW among women and men sick-listed for CMDs in Sweden, also when work-related demands are experienced as the main reason for the sick leave period. It is therefore relevant to include experiences from both domains in studies on RTW or in clinical practice of the same. Furthermore, several of these aspects were described differently by women and men, which highlights the need to consider possible gender differences in relation to RTW, while maintaining attention to individual variations.

## Supplementary Information

**Additional file 1.**

## Data Availability

Since the data is based on transcribed interviews, the data cannot be completely anonymized and can therefore not be shared openly; although names, places and the like have been removed from the transcripts, the participants speak of situations and sensitive experiences within specific contexts and with a level of detail that would pose a risk for the participants to be recognized. The participants have also not given their consent for the interview transcripts to be freely shared. Requests by certified researchers for access to the data should therefore be made to the Research and Data Office at the Karolinska Institute, via rdo@ki.se. If permitted by law and ethical approval, which is decided on a case by case basis, the data can be shared.

## Abbreviations

CMD: Common mental disorders
OHS: Occupational health services
OECD: Organization for Economic Co-operation and Development
RTW: Return-to-work

## References

[1] Swedish Social Insurance Agency (2018). The social Insurance in Numbers 2018, Stockholm.

[2] OECD (2015). Fit mind, fit job. From evidence to practice in mental health and work.

[3] Steel Z, Marnane C, Iranpour C, Chey T, Jackson JW, Patel V (2014). The global prevalence of common mental disorders: a systematic review and meta-analysis 1980–2013. Int J Epidemiol.

[4] Angelov N, Johansson P, Lindahl E, Lindström E-A. Women’s and men’s sickness absence (in Swedish). Uppsala: Institute for Evaluation of Labour Market and Education Policy; 2011. Available from: https://www.ifau.se/globalassets/pdf/se/2011/r11-02-kvinnors-och-mans-sjukfranvaro.pdf.

[5] Fossey EM, Harvey CA (2010). Finding and sustaining employment: a qualitative meta-synthesis of mental health consumer views. Can J Occup Ther.

[6] Henderson M, Glozier N, Elliott KH. Long term sickness absence. BMJ. 2005. 10.1136/bmj.330.7495.802. 10.1136/bmj.330.7495.802 PMC556060 15817531

[7] Vänje A. Gender perspectives on work environment and work organization: a knowledge compilation. (In Swedish). Swedish Work Environment Authority, Report 2013:1. ISSN 1650-3171. Available from: https://www.av.se/globalassets/filer/publikationer/kunskapssammanstallningar/under-luppen-genusperspektiv-pa-arbetsmiljo-och-arbetsorganisation-kunskapssammanstallningar-rap-2013-1.pdf.

[8] Holmgren K, Ivanoff SD (2004). Women on sickness absence—views of possibilities and obstacles for returning to work. A focus group study. Disabil Rehabil.

[9] Verdonk P, de Rijk A, Klinge I, de Vries A (2008). Sickness absence as an interactive process: gendered experiences of young, highly educated women with mental health problems. Patient Educ Couns.

[10] Courtenay WH (2000). Constructions of masculinity and their influence on men's well-being: a theory of gender and health. Soc Sci Med.

[11] Valkonen J, Hänninen V (2013). Narratives of masculinity and depression. Men Masc.

[12] Hultqvist S, Nørup I. Sickness absence and gender in the Nordic countries: what we know and what we do not know. A Literature Review. Nordic Welfare Center; 2016. Available from: http://norden.divaportal.org/smash/get/diva2:1065741/FULLTEXT01.pdf.

[13] Hees HL, Nieuwenhuijsen K, Koeter MW, Bültmann U, Schene AH (2012). Towards a new definition of return-to-work outcomes in common mental disorders from a multi-stakeholder perspective. PLoS One.

[14] Nielsen K, Yarker J, Munir F, Bültmann U. IGLOO: an integrated framework for sustainable return to work in workers with common mental disorders. Work Stress. 2018. 10.1080/02678373.2018.1438536.

[15] Voydanoff P (2002). Linkages between the work-family interface and work, family, and individual outcomes: an integrative model. J Fam Issues.

[16] Mastekaasa A (2000). Parenthood, gender and sickness absence. Soc Sci Med.

[17] Cornelius L, Van der Klink J, Groothoff J, Brouwer S (2011). Prognostic factors of long term disability due to mental disorders: a systematic review. J Occup Rehabil.

[18] Lagerveld S, Bültmann U, Franche R, Van Dijk F, Vlasveld M, Van der Feltz-Cornelis C (2010). Factors associated with work participation and work functioning in depressed workers: a systematic review. J Occup Rehabil.

[19] Blank L, Peters J, Pickvance S, Wilford J, MacDonald E (2008). A systematic review of the factors which predict return to work for people suffering episodes of poor mental health. J Occup Rehabil.

[20] Dellve L, Ahlborg T (2012). Partner relationships and long-term sick leave among female workers: consequences and impact on dimensions of health and return to work. Scand J Caring Sci.

[21] Corbière M, Negrini A, Durand M-J, St-Arnaud L, Briand C, Fassier J-B (2017). Development of the return-to-work obstacles and self-efficacy scale (roses) and validation with workers suffering from a common mental disorder or musculoskeletal disorder. J Occup Rehabil.

[22] Andersen MF, Nielsen KM, Brinkmann S. Meta-synthesis of qualitative research on return to work among employees with common mental disorders. Scand J Work Environ Health. 2012:93–104. 10.5271/sjweh.3257 22025244

[23] Östlund G, Cedersund E, Hensing G, Alexanderson K (2004). Domestic strain: a hindrance in rehabilitation?. Scand J Caring Sci.

[24] Tong A, Sainsbury P, Craig J (2007). Consolidated criteria for reporting qualitative research (COREQ): a 32-item checklist for interviews and focus groups. Int J Qual Health Care.

[25] Kitzinger J (1995). Qualitative research. Introducing focus groups. BMJ.

[26] Bergström G, Lohela-Karlsson M, Kwak L, Bodin L, Jensen I, Torgén M (2017). Preventing sickness absenteeism among employees with common mental disorders or stress-related symptoms at work: design of a cluster randomized controlled trial of a problem-solving based intervention versus care-as-usual conducted at the occupational health services. BMC Public Health.

[27] Hodgins M, MacCurtain S, Mannix-McNamara P (2014). Workplace bullying and incivility: a systematic review of interventions. Int J Workplace Health Manag.

[28] Zigmond AS, Snaith RP (1983). The hospital anxiety and depression scale. Acta Psychiatr Scand.

[29] Glise K, Hadzibajramovic E, Jonsdottir I, Ahlborg G (2010). Self-reported exhaustion: a possible indicator of reduced work ability and increased risk of sickness absence among human service workers. Int Arch Occup Environ Health.

[30] Kitzinger J (1994). The methodology of focus groups: the importance of interaction between research participants. Sociol Health Illn.

[31] Elo S, Kyngäs H (2008). The qualitative content analysis process. J Adv Nurs.

[32] Håkansson C, Ahlborg G (2010). Perceptions of employment, domestic work, and leisure as predictors of health among women and men. J Occup Sci.

[33] Wagman P, Nordin M, Alfredsson L, Westerholm PJ, Fransson EI (2017). Domestic work division and satisfaction in cohabiting adults: associations with life satisfaction and self-rated health. Scand J Occup Ther.

[34] Statistics Sweden. Women and men in Sweden 2018. Facts and Figures. Stockholm; 2018. ISBN: 978–91–618-1658-3. (In Swedish – English summary).

[35] Eva Vingård F. Mental ill health, working life and sickness absence. A knowledge compilation. Swedish Research Council for Health, Working Life and Welfare; 2015. ISBN: 978-91-88561-02-2. Available from: https://forte.se/app/uploads/2015/04/psykisk-ohalsa-arbetsliv.pdf.

[36] Swedish Social Insurance Agency. Sickness absence in mental diagnoses. A study of Sweden's population aged 16–64. Social Insurance Report; 2014:4. ISSN 1654–8574. (In Swedish – English Summary). Available from: http://www.forskasverige.se/wp-content/uploads/Sjukfranvaro-Psykiska-Diagnoser-2014.pdf.

[37] Zapf D (2002). Emotion work and psychological well-being: a review of the literature and some conceptual considerations. Hum Resource Manage R.

[38] Cadge W, Hammonds C (2012). Reconsidering detached concern: the case of intensive-care nurses. Perspect Biol Med.

[39] Lester J (2008). Performing gender in the workplace: gender socialization, power, and identity among women faculty members. Community Coll Rev.

[40] de Vries G, Hees HL, Koeter MW, Lagerveld SE, Schene AH (2014). Perceived impeding factors for return-to-work after long-term sickness absence due to major depressive disorder: a concept mapping approach. PLoS One.

[41] Swedish Social Insurance Agency. Sick leave after immigration. Foreign-born access to and use of sickness benefit is born. Social Insurance Report; 2017:7. ISSN 1654–8574. (In Swedish – English Summary.) Available from: https://www.forsakringskassan.se/wps/wcm/connect/95884afa-4dbf-49d6-8a42-7ad23fcf1803/socialforsakringsrapport-2017-07.pdf?MOD=AJPERES&CVID=.

[42] Shields SA (2008). Gender: an intersectionality perspective. Sex Roles.

[43] Emslie C, Hunt K (2009). ‘Live to work’or ‘work to live’? A qualitative study of gender and work–life balance among men and women in mid-life. Gend Work Organ.

[44] Shirani F, Henwood K, Coltart C (2012). " Why aren't you at work?": negotiating economic models of fathering identity. Fathering..

[45] Swami V (2012). Mental health literacy of depression: gender differences and attitudinal antecedents in a representative British sample. PLoS One.

[46] Englund A-CD, Rydström I, Dellve L, Ahlstrom L (2016). Social support outside work and return to work among women on long-term sick leave working within human service organizations. Appl Nurs Res.

[47] Holmlund L. Return to work: exploring paths toward work after spinal cord injury and designing a rehabilitation intervention. Doctoral thesis, Karolinska Institutet. 2019. Available from: https://openarchive.ki.se/xmlui/handle/10616/46616.

[48] Carlsen B, Glenton C (2011). What about N? A methodological study of sample-size reporting in focus group studies. BMC Med Res Methodol.

[49] Morgan DL (1996). Focus groups. Annu Rev Sociol.

[50] McLafferty I (2004). Focus group interviews as a data collecting strategy. J Adv Nurs.

