# Supplementary material for: Do work- and home-related demands and resources differ between women and men during return-to-work? A focus group study among employees with common mental disorders
Source: BMC Public Health. 2020 Dec 17;20:1914. doi: 10.1186/s12889-020-10045-4 (PMC7745371; doi:10.1186/s12889-020-10045-4)
Supplement: Supplementary file 1 — Additional file 1. [file 12889_2020_10045_MOESM1_ESM.docx]

**Supplementary File 1. Question areas in the interview guide**

*Presentation of the focus group participants*

- Could you tell us a little about yourself?
  - Name, home or working conditions, and so on.

*Home- and work-related demands and how they affect return to work*

- Describe what home- and work-related demands you had during the period before the sick leave.
  - In what way did these demands affect your wellbeing?
- Which home- and work-related demands do you find most difficult to handle?
- How did home- and work-related demands change during your sick leave period?
- In your experience, how do home- and work-related demands affect your return to work?
  - Can you tell us about the home- and work-related demands that constitute the biggest obstacles to return to work?
  - Can you tell us about which home- and work-related resources are supportive for returning to work?

*Experiences of the intervention and their effects*

- Can you tell us about the help/rehabilitation efforts that you received from the occupational health care and which intervention/interventions you received?
- In your experience, were the rehabilitation efforts useful to facilitate return to work?
  - Can you tell us about what was good and what was less good?
- Did you feel that something was missing but which there was a need for?
- Did any of the rehabilitation efforts affect the home- and work-related demands?

*Have the experiences been affected by being a man or a woman*

- In your opinion, would any of the experiences that we have spoken of today differed if you had been of the opposite sex? (For example, would the demands at home have looked different? Proposed rehabilitation efforts? Help received from the workplace?)
  - In what way?

*Can we contact you later if something comes up in the analyses that we would like to ask additional questions about?*

*Is there anything that we haven’t brought up but that you would like to add? Something that feels unclear or that you are thinking about?*

- Give feedback on the discussion.
  - Address points that seemed ambiguous or difficult to understand.
  - Address some key points as a summary.
  - Does this summary agree with what we have talked about?

*Probing questions*

- Encourage further reasoning with questions such as:
  - What do you mean by that?
  - Can you clarify…?
  - Interesting, what do the rest of you think?
  - How does this relate to… demands/resources/return to work/rehabilitation efforts?
